# The Landscape of Neutralizing Monoclonal Antibodies (nAbs) for Treatment and Prevention of COVID-19

**DOI:** 10.1007/s12247-023-09713-w

**Published:** 2023-02-20

**Authors:** Aline de Almeida Oliveira, Diana Praia Borges Freire, Ana Rodrigues de Andrade, Amanda de Miranda Marques, Luciana da Silva Madeira, José Procópio Moreno Senna, Ivna Alana Freitas Brasileiro da Silveira, Beatriz de Castro Fialho

**Affiliations:** Immunobiological Technology Institute, Bio-Manguinhos/Fiocruz, Oswaldo Cruz Foundation, Avenida Brasil, 4.365, NAPA, Manguinhos, Rio de Janeiro, RJ 21040‑900 Brazil

**Keywords:** COVID-19, SARS-CoV-2, Antibody therapeutics, Neutralizing antibody, Technology foresight

## Abstract

**Purpose:**

After nearly 3 years of the COVID-19 pandemic, even though a vast body of knowledge and products (including vaccines and treatments) have been developed and disseminated, the virus is still evolving and new variants arising. Consequently, thousands of lives continue to be lost. Neutralizing monoclonal antibodies (nAbs) are promising drugs that emerged to treat SARS-CoV-2. In the uncertainty of the current situation, there is the question of whether organizations should continue to invest in this technology. To help decision-making in scientifical and pharmaceutical organizations, it is of major importance to monitor the development of products and technologies. Therefore, the aim of this study is analyze the landscape of nAbs for COVID-19.

**Methods:**

The scenario of 473 biotherapeutics focusing on nAbs was evaluated using foresight techniques and a review of literature. Data were obtained from structured and semi-structured databases and processed for treatment, cleaning, consistency, validation, and enrichment.

**Results:**

We identified 227 nAbs and performed an extensive literature review of 16 nAbs in late clinical development, including development technologies, responses to variants of concern (VOCs), manufacturing, and clinical aspects.

**Conclusions:**

Even though the emergence of new VOCs is a threat to the effectiveness of this treatment, demanding constant genomic surveillance, the use of nAbs to treat and prevent COVID-19 will probably continue to be relevant due to excellent safety profiles and the possibility of immediate immunity transfer, especially in patients showing inadequate immunological response to vaccination. Therefore, we suggest that organizations should keep investing in improvements in this technology.

## Introduction

Future predictability for decision-making is not a new issue either in management studies or in firms’ day-to-day process, especially when dealing with technologies and product development. In this regard, scholars and practitioners have developed and tested different tools and techniques to answer a myriad of questions. Turbulent times, such as disease outbreaks, epidemic or pandemic periods, turn decision-making much more complex, especially in areas where knowledge is being generated as the health emergency unfolds and paradigms are not well established [1–3]. This was particularly the case of the COVID-19 pandemic, where high uncertainty has been present since the beginning.

Severe acute respiratory syndrome coronavirus 2 (SARS-CoV-2), a highly pathogenic and transmissible virus, emerged in late 2019 and caused the disease pandemic entitled coronavirus disease 2019 (COVID-19) [4]. By September 2022, more than 620 million people worldwide had been contaminated by SARS-CoV-2, and more than 6 million died [5]. The race for prevention started in February/2020, when there were 21 vaccine projects in pre-clinical and clinical development, according to a publication by our group [6]. In addition to prevention, much research has been done regarding treatments, including biological and synthetic drugs. To accelerate the availability of drugs for the treatment of COVID-19 at the beginning of the pandemic, there was a significant investment in the repositioning of medicines whose clinical efficacy and safety had already been demonstrated for other diseases. Unfortunately, the repositioning of drugs for COVID-19 has brought few significant results in treating mild cases and in the early stages of the disease. The best results occurred in the context of hospitalized patients, with emphasis on the following drugs: dexamethasone, remdesivir, tocilizumab, sarilumab, and baricitinib [7–10].

Recently, two new synthetic antivirals of oral administration, molnupiravir and nirmatrelvir-ritonavir, were approved for emergency use in some countries and are indicated for the treatment of COVID-19 in its initial phase. Both showed good results in clinical studies with some possible limitations [11, 12]. Hence, there is concern about a possible viral mutagenic effect of molnupiravir in immunosuppressed patients as they have reduced viral clearance. More data on safety regarding use in childbearing age are still needed since an animal study has demonstrated teratogenesis. Regarding Nirmatrelvir/Ritonavir, the limitations are related to the interaction with other drugs that use the CYP3A pathway, requiring careful medication conciliation to avoid loss of antiviral activity due to increased drug metabolism, in addition to the necessary adjustment for renal function [13, 14].

Even with the approval of some drugs for emergency use and of vaccines against COVID-19 (more than 10 billion doses of vaccines have already been applied), thousands of lives continue to be lost [2], and the disease is not yet eradicated. One of the reasons is that vaccination coverage is asymmetric. Although on average of about 70% of the world population is vaccinated, in Africa, most countries have a vaccine coverage below 40% [5]. In addition, it is not yet known how the effectiveness of vaccines will vary over time and how the variants of concern (VOCs) will interfere with vaccine-mediated protection.

Thus, there is still a need to develop new treatments and complementary tools to prevent the spread of COVID-19 in unvaccinated or immunocompromised people who cannot generate an adequate immune response. Despite great worldwide efforts, the limits of knowledge on the biological mechanisms of COVID-19 regulation made drug discovery difficult [7].

In this context, this prospective study analyzes the scenario of treatment development for SARS-CoV-2, focusing specifically on a promising emerging class of drugs, the therapeutic neutralizing antibodies (nAbs), which are antibodies that protect the host cell from pathogens by neutralizing or inhibiting its biological effect. In viral diseases, they may be applied to block interactions of the viral envelope with the host cell receptor or inhibit the release of the viral genome. Even though the only approved nAb was palivizumab, several clinical trials for a wide range of viruses (HIV, Ebola, MERS-CoV, CHIKV, and SARS-CoV) were ongoing when the COVID-19 pandemic started. Since then, anti-SARS-CoV-2 nAbs were widely characterized in pre-clinical studies, were also the object of randomized clinical studies, controlled in different scenarios, and approved for emergency use by regulatory agencies such as FDA, EMA, and ANVISA [8, 15–20].

Since the beginning of the pandemic, several initiatives aimed at identifying products under development and their stage of advancement have been launched by research groups, such as London School of Hygiene & Tropical Medicine [21] and Milken Institute [22], and journalistic/information companies such as The NYT COVID Tracker [23], in addition to scientific articles published. Although vaccines have been developed and deployed, diagnostics improved, new treatments developed and registered, and a vast body of knowledge has been developed and disseminated, and access to vaccines, diagnostics, and treatments is still highly inequitable. At the same time, the virus is still evolving and new variants emerging. Therefore, the scenario remains unstable, justifying the major importance to keep monitoring the development of products and technologies.

Then, the main purpose of the present study is to analyze the current scenario of nAbs in COVID-19 and identify future trends, aiming to help the decision-making on technological investments in scientific and pharmaceutical organizations. As far as we know, there are no published articles regarding this subject combining a review of the literature and a technology of foresight analysis.

## Methodology

A systematic technology foresight (TF) study focusing on nAbs anti-SARS-CoV-2 was carried out to analyze the scenario including, among others, their status and technological attributes. It is noteworthy that some of these products had already been approved for emergency use by worldwide regulatory authorities.

The methodology was based on a multidisciplinary foresight network formed at the beginning of the COVID-19 pandemic and on the 5W2H concept (Who? Where? What? Why? When? How? How much?). This network comprises foresight working groups (FG) and discussion groups (DG). The FG is responsible for collecting, processing, and analyzing the data obtained, and the DG for studying the scientific literature on products and technologies and analysis of the data. Professionals from different background knowledge areas, such as Agricultural Sciences, Biological Sciences, Health Sciences, Exact and Earth Sciences, Applied Social Sciences, and Engineering, formed these groups. It is important to highlight that these specialists work in the pharmaceutical biotechnology industry and are experienced in the development, technology transfer, and/or production of biopharmaceuticals, vaccines, and diagnostic reagents.

Due to the health emergency, the following main initial questions guided the research: “What are the biological drugs and vaccines in development for COVID-19?,” “What are their technological trends?,” “What is their stage of development?,” “What is their molecular target?,” “What is their mode of action?,” “Which organizations are involved?,” and “What is their dosage and route of administration?.” Data collection, preparation, and analysis were based on the information collected in private databases, mainly AdisInsight^®^, and public databases, predominantly Clinical Trials Gov (CTG)^®^, International Clinical Trials Registry (ICTRP)^®^, Antibody Society’s COVID-19 Biologics Tracker [17], and regulatory agencies (FDA [8], Anvisa [18], and EMA [16]) as summarized in Table 1.

**Table 1:** Procedures for building the COVID-19 antibody therapeutics database

**Step 1**	**Data collection**	Source: AdisInsight^®^, CTG^®^, and ICTRP^®^.
		- Search for AdisInsight^®^: Indication “coronavirus” and “cytokine-related syndrome” - Search for CTG^®^: Condition or disease (SARS-CoV-2 OR 2019-nCoV OR 2019 novel coronavirus OR severe acute respiratory syndrome coronavirus 2 OR coronavirus) - Search for ICTRP^®^: “Download COVID-19 trials csv format”
		Period: February/2020 to September/2022
		Method: (1) The product pipeline for coronavirus, including projects and technologies, was collected from AdisInsight^®^. (2)The clinical trials related to the products were collected from CTG^®^ and ICTRP^®^. (3) Scientific articles (published and preprints), press releases, and specialized websites from regulatory agencies, COVID-19 Biologics Tracker, and organizations involved in R&D related to the products were consulted for data preparation.
**Step 2**	**Data preparation**	Cleaning: The identification of duplicates, incomplete, and/or inaccurate records.
		Treatment and qualitative validation: The comparison of these data with others not originally included in the consulted databases was performed manually, aiming to transform and initiate enrichment for knowledge generation. Data were classified into several categories: technologies, modes of action, development stages, and status to facilitate data analysis.
**Step 3**	**Data analysis**	Qualitative and multidisciplinary data analysis was carried out based on the data and information made available during the research by a DG, thus contributing to the enrichment of the results.
**Step 4**	**Data presentation**	Data presentation was performed by data classified and characterized in different groups to generate tables in Microsoft Excel^®^ and charts using visualization software (PowerBI Desktop^®^ and Vantage Point^®^), allowing better visualization of the results and analysis.
**Step 5**	**Update and maintenance**	New data are being collected and analyzed frequently by FG and DG.

*CTG*^®^ Clinical Trials Gov, *ICTRP*^®^ International Clinical Trials Registry, *R&D* research and development, *FG* foresight working groups, *DG* discussion groups

All collected data underwent a procedure that included the identification of duplicate, incomplete, or inaccurate records. After this first preparation step, the treatment and qualitative validation of the consistency of the data obtained was performed. The FG compared these data with others not initially included in the consulted databases, aiming to transform and initiate enrichment for knowledge generation.

After organizing the information by the FG, a qualitative and multidisciplinary analysis was carried out based on the data and information made available during the evaluation by the DG, thus contributing to the enrichment of the results. At this point, we were interested in finding out what are the prospects for this market and expectation of the life cycle of these products, considering the cost of treatment, the wide application of vaccines and the emergence of new variants.

Aiming at refining understanding and enabling broad coverage of scenario analysis, the data were classified and characterized in different groups, using the Microsoft Excel^®^, Vantage Point^®^, and PowerBI Desktop^®^ software to improve analysis and the discussion of information. It is noteworthy that the same product may be currently in different R&D stages in different countries, with different goals (for example, prevention or treatment), or with different formulations. Therefore, it may appear as a duplicate in the database and figures. To minimize the duplication of products, the records related to finished phases and unknown status phases were excluded using filters. The phases of development were classified as: preclinical (R&D), early clinical development (phase I, II, or I/II), late clinical development (phase II/III or III), preregistration (submitted to registration), or commercially available (products registered or emergency use registered). Furthermore, since information on the research and preclinical phases is less accurate, it is not included in all analysis.

## Results and Discussion

### Analysis of Biological Products Scenario for COVID-19

**Fig. 1:**
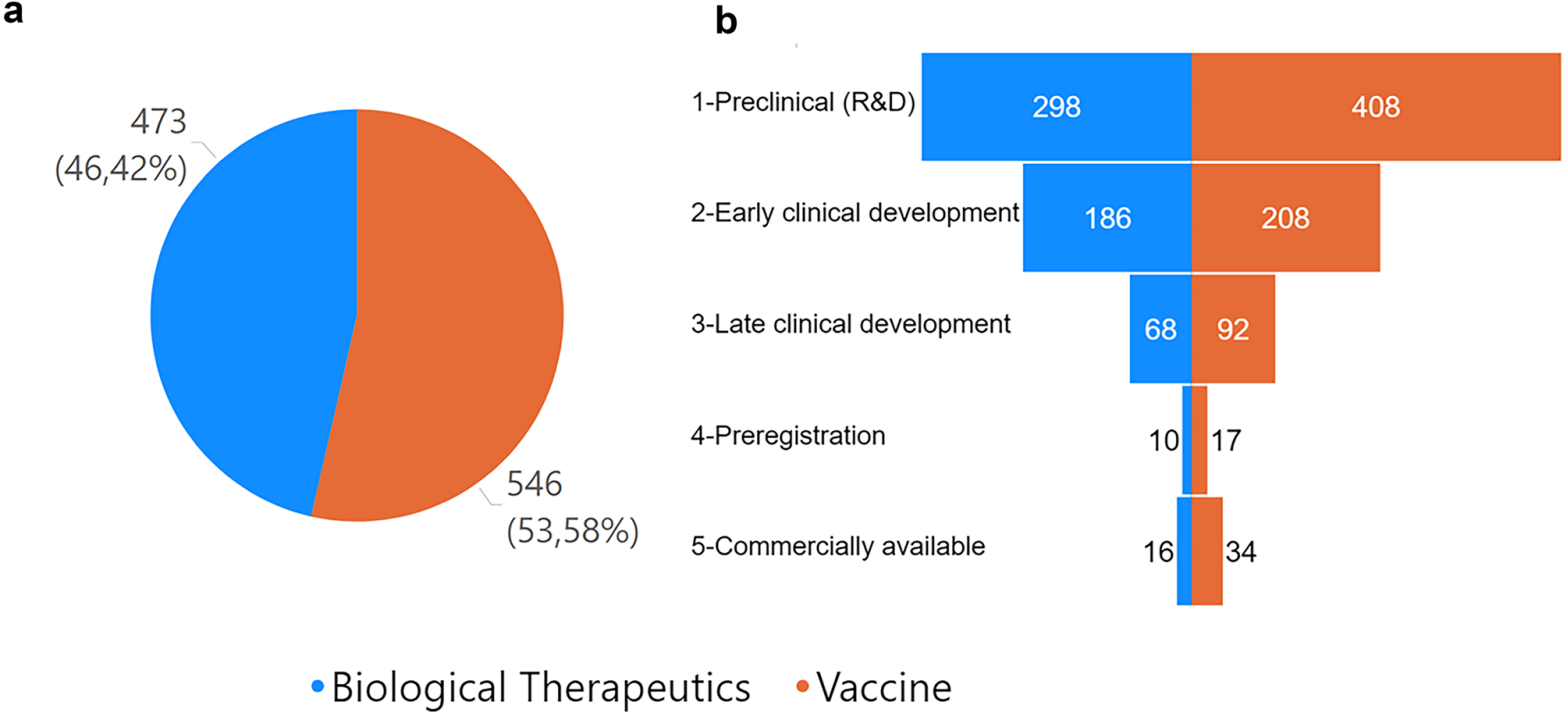
The number of vaccines (orange) and biological medicines (blue) of the database is presented in (**a**). The number of records of development phase by product category is presented in (**b**)

According to the database enriched as described above, more than 1000 records of products were found in different stages of development to treat or prevent COVID-19. Among them, 473 biological therapeutic products and 546 vaccines in different stages of development were found (Fig. 1).

**Fig. 2:**
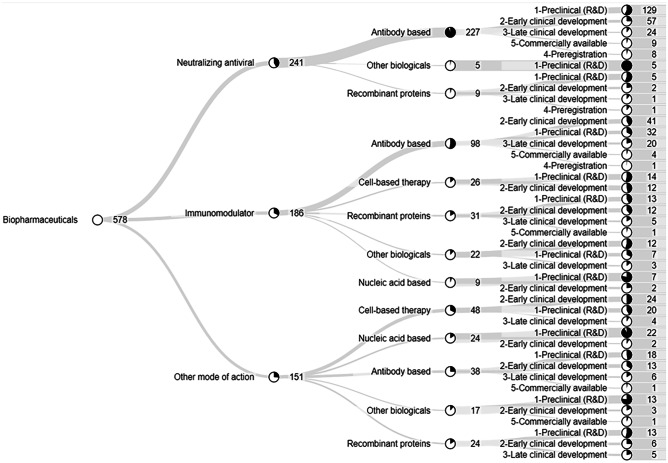
Classification of biopharmaceuticals by mode of action followed by technology niche and development phase

Biopharmaceuticals were further characterized as seen in Fig. 2. Most biopharmaceuticals in development had their modes of action classified as immunomodulators (186), or neutralizing antivirals (241) as observed in Fig. 2. Regarding the technological niche, most products in all modes of action were antibody based, including 227 of the neutralizing mode of action.

Several articles have already been published reviewing anti-SARS-CoV-2 nAbs from a clinical or technological point of view [15, 19, 24–28]. However, only Yang et al. [28] presented a methodology for data processing based on an algorithm. They found 217 antibody-based products, 89 of which presented the spike protein as molecular target. At the time of that publication, there were only 8 nAbs in clinical trials, and none were yet approved for emergency use. Besides the use of data cleaning and treatment methodology, the present work makes a literature review on various aspects of nAbs for COVID-19.

It is important to note that the process carried out in the present study is not based on the use of an automated algorithm to generate the data but on the collection of data from a search strategy in a structured private base and extensive data treatment and discussion with a multidisciplinary group of experts. This allows for data curation and double verification during the processing and enrichment processes. However, it should be noted that it is not always possible to find all the necessary information during the data preparation stage. Quite often, institutions involved in research and development publish information with a certain degree of imprecision, whether in their internet domains or press releases. Among the challenges, we highlight characterizing the technological route and details of the technology used, the removal of duplicates when a product in research phase receives a different identification code when it advances in phase, or when there is no update of the product advancement [29]. All data was collected in the period between February/2020 and September/2022.

### Neutralizing Antibodies (nAbs) for the Treatment of COVID-19

Classically, antiviral monoclonal antibodies can act by two main mechanisms—one is the direct activity on the pathogen (neutralizing activity) and the other through the recruitment of effector functions of the host’s immune system. The neutralizing activity occurs by the direct binding of the antibody with a specific viral epitope, sufficient to neutralize the viral particle. This form of activity is considered independent of host cells or molecules [20, 26, 28, 30, 31]. Animal models have shown the effectiveness of nAbs in reducing symptoms and viral replication, in addition to its prophylactic effect, showing potential for lowering viral transmission [32–37]. Clinical trials have demonstrated a good safety profile [38–45] and some promising, but preliminary, results of efficacy in the prevention and treatment of COVID-19 [38, 42–45]. Recently, encouraging real-life data have been published for the use of nAbs in preventing hospitalization and mortality in outpatients [46, 47].

**Fig. 3:**
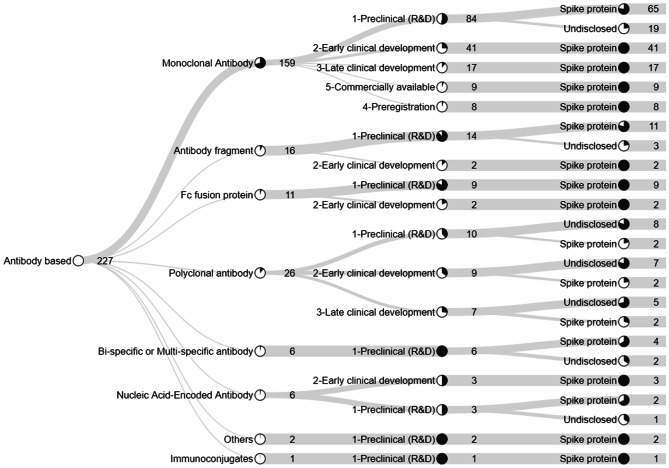
Technology details and development phases of neutralizing antibodies

In the current study database, there were 227 nAbs (Fig. 3) in different development stages. Most products were monoclonal antibodies (159). Other antibody technologies, such as fragments, bi-specific and multi-specific, and immunoconjugates were rare and in earlier development phases; 26 polyclonal antibodies were present in our database, most of them from convalescent plasma. Passive immunization with convalescent plasma involves transfusing the acellular part of blood from patients who have recovered from an infection to persons who are infected or at risk of infection. Plasma donors are presumed to have developed antibodies against the pathogen [13, 19].

Some studies regarding convalescent plasma showed benefits and others, including metanalysis, were inconclusive. This therapy requires multiple factors to be successful, specially timing and quality control, and there are associated risks of incompatibility or infections. Other sources of polyclonal antibodies were also evaluated for COVID-19 treatment, such as general and specific intravenous immunoglobulin (IVIg), hyperimmune equine serum therapy, among others. While there appears to be a potential benefit for these approaches, there are some downsides and limitations to its use, such as batch-to-batch variation and supply issues [19]. Also, the great number of VOCs imposes a particular challenge to on-time convalescent plasma development for adequate use. National Institute of Health (NIH) and Infectious Diseases Society of America (IDSA) guidelines on the treatment and management of patients with COVID-19 strongly recommend against its use in hospitalized patients and conditionally recommend its use for immunosuppressed patients without other therapeutic options [13].

**Fig. 4:**
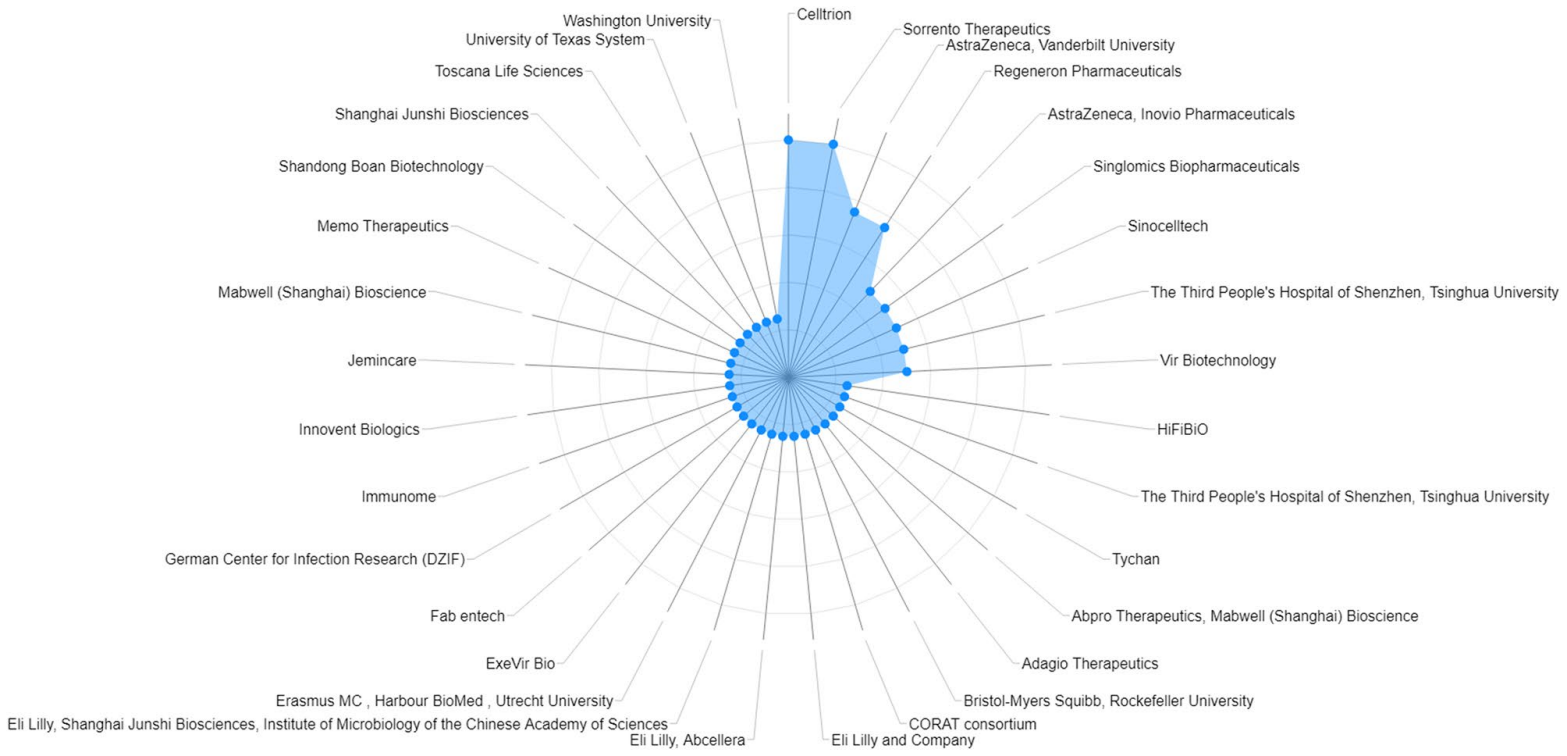
Organizations involved in development of nAbs. The circles represent the number of nAbs in clinical or more advanced phases

**Fig. 5:**
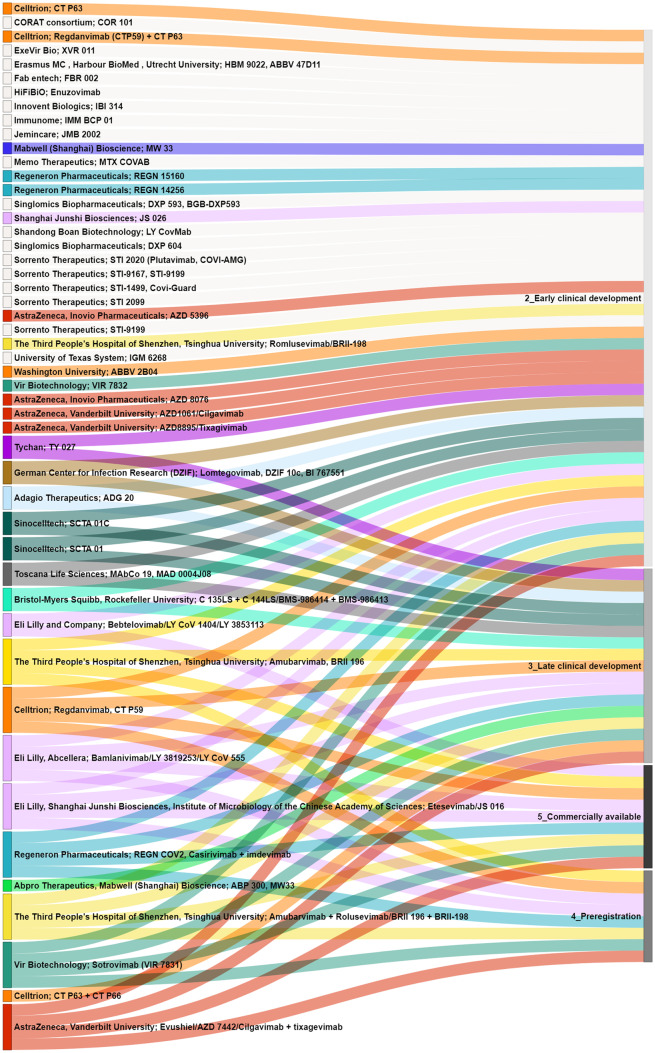
Sankey chart of nAbs (organizations and products names) by clinical phase. Each organization involved in late clinal trials was highlighted in a different color, and the lines are coming from the name of products and organizations to reach the development stage at the right in different shades of gray

Around 30 organizations involved in the clinical development of nAbs were identified (Fig. 4). The companies and drug names were evaluated by development phases (Fig. 5). Several organizations had different products in different phases, such as Celltrion (CT-P59, CT-P63, and CT-P66), Regeneron Pharmaceutics (casirivimab, imdevimab, REGN15160, and REGN14256), Vir (sotrovimab and Vir 7832), AstraZeneca (cilgavimab, tixagevimab, AZD 5396, and AZD 8076). A summary of the characteristics of the 16 nAbs found in the late clinical development is presented in Table 2.

**Table 2:** Main nAbs in late clinical development and/or regulatory phase

**Name (alternate name(s))**	**Developers**	**Dosage/route of administration**	**Main clinical trials**	**Development phase**
ADG20 (ADG2/ADI-55688/adintrevimab)	Adagio Therapeutics/Biocon Biologic	300 mg IM	NCT04805671—phase II/III NCT04859517—phase II/III	Phase II/III
AZD 7442 (AZD8895/COV2-2196/tixagevimab + AZD1061/COV2-2130/cilgavimab)	AstraZeneca/Vanderbilt University	300 mg + 300 mg/IM (pre-and post-exposure prophylaxis) 600 mg/IM (early treatment)	NCT04507256—phase I NCT04896541—phase I NCT04625725—phase III NCT04315948—phase III NCT04723394—phase III NCT04518410—phase II/III NCT04501978—phase III NCT04625972—phase III	Phase III Emergency use approved by FDA and Anvisa
BRII-196 (amubarvimab/P2C-F11) + BRII-198 (romlusevimab/P2B-1G5)	The Third People’s Hospital of Shenzhen, Tsinghua University/Alfacell Corporation/Brii Biosciences	1000 mg + 100 mg/IV (late treatment)	NCT04479644—phase I NCT04479631—phase I NCT04691180—phase I NCT04770467—phase II NCT04787211—phase II NCT04518410—phase II/III NCT04501978—phase III	Phase III Emergency use approved by China’s NMPA
C135LS/C (BMS-986414) + C144LS (BMS-986413)	The Rockefeller University/Bristol Myers Squibb	2 × 200 mg/SC + 2 × 200 mg/SC	NCT04700163—phase I NCT04518410—phase II/III	Phase II/III
CT-P59 (regdanvimab)	Celltrion	40 mg/kg/IV (early treatment)	NCT04525079—phase I NCT04593641—phase I NCT04602000—phase II/III	Phase II/III Emergency use approved by EMA/ Anvisa / South Korea /Canada
LY-CoV016 (JS-016/CB6/LY3832479/etesevimab) + LY-CoV555 (LY3819253/bamlanivimab)	Eli Lilly and Company; Institute of Microbiology of the Chinese Academy of Sciences; Shanghai Junshi Biosciences + Abcellera; Eli Lilly and Company; National Institute of Allergy and Infectious Diseases	700-mg Bamlanivimab IV/1.400 mg of Etesevimab IV	NCT04441931—phase I NCT04611789—phase I NCT04427501—phase II/III NCT04497987—phase III	Phase III Emergency use approved by FDA/EMA/ Anvisa^a^
LY-CoV1404 (LY3853113/bebtelovimab)	Eli Lilly and Company/ Abcellera	175 mg/IV	NCT04634409—phase II	Phase II Uso emergencial aprovado pelo FDA
MAbCo 19 (MAD 0004J08)	Toscana Life Sciences, AchilleS Vaccines	100 mg ou 400 mg/IM	NCT04932850—phase I NCT04952805—phase II/III	Phase II/III
REGN-COV2 (REGN10933/casirivimab + REGN10987/imdevimab)	Regeneron/Roche	600 mg + 600 mg IV or SC	NCT04617535 NCT05092581—phase Ib NCT05081388—phase I/II/III NCT05074433—phase III NCT04992273—phase IIa NCT04852978—phase II NCT04425629—phase I/II/III	Phase III Emergency use approved by FDA/EMA/ Anvisa^b^
SCTA 01	Sinocelltech	IV/50 mg/kg	NCT04483375—phase I NCT04644185—phase II/III NCT04683328—phase II/III NCT04709328—phase II/III	Phase II/ III
TY 027	Tychan	IV/1.500 mg or 2.000 mg	NCT04429529—phase I NCT04649515—phase III	Phase III
VIR 7831 (S309/sotrovimab)	Vir/GSK	500 mg/IV	NCT04545060—phase II/III NCT04779879—phase II NCT04913675—phase III	Phase II/III Emergency use approved by FDA/EMA/ Anvisa^b^

Own elaboration based on the group’s database

*IM* intramuscular, *IN* intranasal, *IV* intravenous, *SC* subcutaneous

^a^These products have been approved for emergency use but have been revoked by the FDA, EMA, and Anvisa due to the high frequency of the Omicron variant

^b^These products have been approved for emergency use but have been revoked by the FDA* due to the high frequency of the Omicron variant or subvariants. DZIF 10c and ABP 300 (MW33) were in late clinical development, but there was not enough available information to be displayed in the table

Virtually, all nAbs for treatment of COVID-19 target the spike (S) protein of the virus (Fig. 3), blocking the interaction of the S protein trimer with the angiotensin-converting enzyme 2 (ACE2) receptor in host cells. Briefly, the S1 subunit initiates the process of virus invasion by mediating the binding of the N-terminal domain (NTD) of hACE2, while the S2 subunit directs the fusion of viral and cellular membranes. The S1 subunit is composed of two domains: NTD and receptor-binding domain (RBD), specifically the receptor-binding motif (RBM) that interacts with hACE2 in the segment of residue 446–505. The three RBDs undergo a conformational equilibrium shift like a hinge from a closed pre-fusion “down” state to a fusion-prone or “up” state. nAbs isolated from convalescent SARS-CoV-2 patients often recognize RBD of spike protein, which presents high flexibility [48–51].

Neutralization has long been the principal mechanism of action of antibodies during viral infections, but several studies have shown that Fc-mediated effector functions play a significant role in the antibody response to viral infections. The importance of Fc interactions varies between antibodies and may be influenced by epitope location, binding affinity, breadth of reactivity, neutralization potency, and time of administration [52].

Another type of action of antiviral monoclonal antibodies is through the recognition of the Fc portion by cells or molecules of the immune system, generating the following effector functions: (i) opsonization, where the antibody binds to the pathogen’s receptors, attracting immune cells (neutrophils and macrophages) to phagocytize and destroy them; (ii) complement-dependent cytotoxicity (CDC), which induces lysis through the membrane attack complex (MAC); (iii) antibody-dependent cell-mediated cytotoxicity (ADCC), a defense mechanism mediated by effector cells of the immune system that actively lyses a target cell whose membrane surface has been coated with specific antibodies [30].

Several strategies have been used for the discovery of nAbs for COVID-19 treatment. The majority are based on technologies for the generation of human antibodies, such as phage display, humanized transgenic mice, and the identification and selection of B lymphocytes from samples of convalescent SARS-CoV2 patients as shown in Table 3. Unlike this pattern, some nAbs were discovered from samples of patients convalescent for SARS-CoV, such as sotrovimab and ADG20, presenting a good range of responses against several viruses of the Sarbecovirus genus [27, 33, 53–55].

**Table 3:** Individual characteristics of neutralizing antibodies in late clinical development and/or regulatory phase (phase 2/3 and phase 3)

**Name** **(Alternate name(s))**	**Fc modifications**	**Technology of discovery**	**Antigen binding and neutralizing ability**	**References**
ADG20 (ADG2/ADI-55688/adintrevimab)	half-life ext. (?)	Obtained from B lymphocytes of SARS-CoV patient (2003); in vitro affinity maturation	1 ng/mL (IC50)/430 pM (Kd)	[53, 55]
AZD 7442 (AZD8895/COV2-2196/tixagevimab + AZD1061/COV2-2130/cilgavimab)	TM (silencing the Fc activity) /YTE (serum half-life)	Obtained from B lymphocytes of COVID-19 convalescents	AZD8895: 9 ng/mL (IC50)/2.8 pM (Kd) + AZD106: 32 ng/mL (IC50)/13.0 pM (Kd)	[58, 62–64]
BRII-196 (amubarvimab/P2C-F11) + BRII-198 (romlusevimab/P2B-1G5)	YTE (serum half-life)	Obtained from B lymphocytes of COVID-19 convalescents	3 ng/mL (IC50)/2120 pM (Kd)	[57, 65, 66]
CT-P59 (regdanvimab)	WT?	Obtained from B lymphocytes of COVID-19 convalescents	8.4 ng/mL (IC50)/27 pM (Kd)	[33, 67, 68]
C135LS/C (BMS-986414) + C144LS (BMS-986413)	LS (serum half-life)	Obtained from B lymphocytes of COVID-19 convalescents	C144: 2.55 ng/mL (IC50)/18,000 pM (Kd) + C135: 2.98 ng/mL (IC50)/6000 pM (Kd)	[69, 70]
LY-CoV016 (JS-016/CB6/LY3832479/etesevimab) + LY-CoV555 (LY3819253/bamlanivimab)	LY-CoV016— LALA (silencing of the Fc activity) LY-CoV555—WT	Obtained from B lymphocytes of COVID-19 convalescents	LY-CoV016 13 ng/mL (IC50)/660 pM (Kd) + LY-CoV555 12 ng/mL (IC50)/1450 pM (Kd)	[39, 42, 43, 53, 70, 71]
LY-CoV1404 (LY3853113/bebtelovimab)	WT?	Obtained from B lymphocytes of COVID-19 convalescents	6.4 ng/mL (IC50)/75 pM (Kd)	[47, 72]
MAbCo 19 (MAD 0004J08/J08)	LS (serum half-life) LALA-PG (silencing the Fc activity)	Obtained from B lymphocytes of COVID-19 convalescents	3.9 ng/mL (IC100)/20 pM (Kd)	[73, 74]
REGN-COV2 (REGN10933/casirivimab + REGN10987/imdevimab)	WT	Obtained from humanized mice (Velocimmune) and B lymphocytes from COVID-19 convalescents	REGN10933: 5.61 ng/mL (IC50)/41 pM (Kd) + REGN10987: 6.31 ng/mL (IC50)/42 pM (Kd)	[40, 58–60, 75]
STA 01 (HB27)	LALA (silencing the Fc activity)	Obtained from mice immunized with SARS-CoV-2 RBD	6 ng/mL (IC50)/220 pM (Kd)	[76, 77]
VIR 7831 (S309/sotrovimab)	LS (serum half-life)	Obtained from B lymphocytes of SARS-CoV patient (2003)	79 ng/mL (IC50)/210 pM (Kd)	[35, 44, 78]

*AV* authentic virus**,** *IC*_*50*_ half-maximal inhibitory concentration, *NA* not available

The nAbs-binding affinity is usually measured by Kd determination using surface plasmonic resonance (SPR) or microcalorimetry and by ACE2 competition assay by ELISA or other immunoassays, while the evaluation of the binding structure of the nAb to the trimeric S protein is performed by X-Ray crystallography and cryo-microscopy. Neutralizing activity is evaluated in an in vitro assay with pseudovirus, using different cells, and in a microneutralization assay (PRNT) with an authentic virus in Vero cells to determine the IC50 [56, 57]. Table 3 presents the Kd and IC50 of the 16 nAbs in an advanced stage of development.

Regarding the preclinical phase, different animal models have been used to analyze the neutralizing assay in vivo such as Rhesus or Macaca mulatta. However, severe acute human disease is only evaluated using golden Hamster, which presents clinical manifestations such as rapid weight loss, high viral load, and severe lung pathology. In addition, the transgenic mice model (hACE2) allows the study of the interactions between the virus and the receptor [55, 58–60].

The development of these nAbs, from the discovery of SARS-CoV-2 to clinical trials, was extremely rapid, taking less than a year in many cases. The virus was identified in China in December 2019, and the first neutralizing antibody was approved for emergency use by the FDA in November 2020 [8]. This speed was only possible due to a combination of recent technological advances, emphasizing the high-throughput methodologies for discovery and high productivity of cell lines for process development. In addition, the strategies of acceptance of a greater degree of risk in the business model and increase in costs by the pharmaceutical industries, without risking the quality and safety of the products, along with carrying out several steps concurrently and leaving the optimization of processes for after clinical trials, also helped to accelerate the development of anti-SARS-CoV-2 nAbs [56, 57, 61]. Many of these strategies have already been successfully applied to accelerate the development of vaccines against COVID-19 [6].

An ideal therapeutic antibody against SARS-CoV-2 would be able to resist viral escape, present activity against several viruses of the Sarbecovirus genus, and, finally, be highly protective through viral neutralization and effector functions [53, 55]. The following sections will address these and other interesting features for a good clinical response.

### Antibody Engineering of nAbs Anti-SARS-CoV-2

Different strategies to modify the Fc region of antibodies were used in several nAbs intended to treat SARS-CoV-2 infections to change certain characteristics of the molecule. A desired feature of monoclonal antibodies used for this disease is its wide distribution in tissues and long plasma half-life, allowing for greater coverage throughout the infectious process. The modifications applied to the most advanced nAbs are presented in Table 3.

Monoclonal antibodies in IgG format characteristically have a half-life of approximately 20 days [30]. Some groups have developed antibodies with an even longer plasma half-life, despite this characteristic. Dall Acqua et al*.* demonstrated that a triple mutation in the Fc region of IgG (M252Y/S254T/T256E (YTE)) could increase binding to the FcRn receptor, improving the plasma half-life of an anti-RSV (respiratory syncytial virus) antibody as well as the bioavailability in the lungs [79]. LS mutations (M428L/N434S), described by Zalewsky et al. 2010, have also been shown to increase the plasma half-life of nAbs [80].

As shown in Table 3, sotrovimab and MabCo 19 present an LS mutation in the Fc region, which increases binding affinity to the FcRn receptor by replacing two amino acids in the Fc domain, thus increasing half-life and pulmonary bioavailability [35, 44, 52, 73, 74, 78, 80]. The AstraZeneca’s [58, 62–64] and BRII’s cocktails [57, 66] were optimized by the triple YTE mutation for half-life extension.

In addition to the increase in plasma half-life, another frequent change in the Fc portion of anti-SARS-CoV-2 nAbs is to reduce its effector function. One of the most likely reasons that could justify such a modification is that, although the recruitment of the effector immune system can also act in the elimination of SARS-CoV-2, there is a concern that ADE (antibody-dependent enhancement) can activate viral propagation and generation of cytokine storm [32]. ADE can occur in two different ways. In the first possibility, specific antibodies could enhance infection by viral uptake and replication in immune cells expressing cell receptors. The other possible way would be the activation of effector functions mediated by the Fc region or the formation of an immune complex [81].

For this purpose, some antibodies have been developed with alterations in the constant region (Fc) of IgGs, with mutations L234A and L235A (LALA mutations), to silence the recruitment of the immune system [81]. The LALA mutation was applied to the anti-SARS-CoV-2 antibodies etesivimab [38] and STA 01 [76, 77] (Table 3). The MabCo 19 antibody, in addition to LALA, incorporates a P329G mutation to further silence effector function [74]. This mutation was previously described and named LALA-PG, which eliminates binding and complement fixation, in addition to ADCC [76]. Another type of modification used for this purpose is TM (triple mutant—L234F/L235E/P331S) [82] applied to AstraZeneca [17, 58] antibodies (Table 3).

Although several nAbs that are being developed present alterations to prevent ADE, studies carried out to evaluate severe infections by COVID-19 showed that there is no definitive evidence of ADE occurrence in SARS-CoV-2 infections [81]. In contrast, the number of experimental evidence from animal models demonstrating that Fc and Fc**γ** interactions are essential for the antiviral activity of anti-SARS-CoV-2 monoclonal antibodies (mAbs) is growing, and the loss of Fc-interacting capacity is associated with reduced antiviral activity in vivo [25, 83].

Furthermore, Ravtech et al*.* suggest that engineering antibodies to increase the binding capacity to Fc**γ** may improve the therapeutic and prophylactic efficacy of anti-SARS-CoV-2 nAbs in animal models, thus using the GAALIE mutations (G236A/A330L/I332E) to improve effector function [83]. This modification is intended to enhance dendritic cell maturation and induction of CD8 + T cell response [25, 83]. In this sense, according to our database, VIR/GSK is investing in another mAb developed from S309, known as VIR-7832, which has a modification of GAALIE (modification of 3 amino acids), which increases binding to Fc**γ** IIa and Fc**γ** IIIa receptors, decreases the affinity for Fc**γ** IIb, and is in phase 2 of clinical study [35].

### Production of nAbs for COVID-19

The treatment involving mAbs requires the use of high doses, as shown in Table 2. This matter, combined with the complex production process, the use of cell culture platforms, and upstream and downstream systems, in addition to the necessary storage requirements, result in an expensive final cost of current therapeutic monoclonal antibodies available on the market. This high cost makes it difficult to use mAbs in low-income countries, especially those that do not have biotechnology institutions for their production [61, 84].

There are few publications about production strategies for anti-SARS-CoV-2 mAbs, and there are big challenges regarding bioprocess optimization and scaling up to generate enough amounts of active pharmaceutical ingredient (API) to meet the world population’s needs. In this sense, the Chinese hamster ovary (CHO) cell platform offers good yields and has been the most widely used for the manufacturing of therapeutic mAbs [61]. Another difficulty in the case of nAbs cocktails would be the need to have multipurpose factories. Even more relevant, it is necessary to discuss the distribution of manufacturing plants in the world, such as in Latin America and Africa, to meet the world’s demand [50].

Since mRNA vaccines have been widely applied for many diseases, including the COVID-19 vaccine, a very promising approach to mitigate the cost of nAbs would be the direct administration (delivery) of synthetic nucleic acids (DNA or RNA) encoding monoclonal antibodies. These approaches use the host as a biological factory to produce the antibodies, eliminating bioprocess steps and providing significant advantages over the traditional process of producing and administering therapeutic antibodies [84, 85].

DNA systems (pDNA-mAbs) are based on the direct cloning of antibody sequences into plasmid vectors for release into host cells. These vectors can encode large and complex proteins such as antibodies. The thermal stability of DNA allows it to be stored at room temperature for long periods of time [85]. The mRNA systems (mRNA-mAbs) rapidly express antibodies, as they do not require the steps of DNA to RNA processing. The lipid nanoparticle allows the release of mRNA inside the cells, transferred directly to the ribosomes for translation in the cell cytoplasm, thus resulting in a fast and efficient release of the protein of interest (in this case, nAbs) [84, 85].

Several studies have demonstrated efficacy in murine and non-human primate models, using DNA and mRNA mAbs against dengue virus, influenza A and B, Ebola, Zika, rabies, and HIV; some of them have reached clinical trials [85]. Regarding SARS-CoV-2, there are some promising preclinical results with an expression of neutralizing antibodies in the lungs through the intranasal application of self-replicating mRNA [86]. In our database, we found 6 nAbs encoded by nucleic acid, 3 of them in the early clinical phase (Fig. 3). AstraZeneca is one of the companies investing in nucleic acid-encoded antibody in association with Inovio Pharmaceuticals (Fig. 5).

### Development Strategies to Respond to Variant of Concerns (VOCs)

VOCs show increased transmissibility, virulence, and/or reduced effectiveness of control measures. Due to the potential of VOCs to decrease the protective immunity effect, several mAbs with potent neutralizing activity have been studied against the different variants of SARS-CoV-2, mainly those in clinical studies or approved for emergency use. The VOCs currently described by the World Health Organization (WHO) are Alpha (B.1.1.7), Beta (B.1.351), Gamma (P.1), Delta (B.1.617.2), and, more recently, Omicron (BA1; BA.2; BA.3; BA.4, and BA.5) [87].

The speed of variants’ emergence demonstrates the need for genomic surveillance of the circulating virus to define the use or discontinuation of drugs, including combinations of mAbs that bind to different epitopes [25, 60, 88, 89]. It was proposed that the combination of two or more neutralizing antibodies in a cocktail that binds to different targets or epitopes of the S protein increases the neutralization potential and may prevent virus variants from being resistant to treatment compared to selective pressure with the use of a single antibody. According to this proposal, 5 cocktails were found in our database that were in advanced clinical development (Table 2).

An example of a successful cocktail was developed by Regeneron Pharmaceuticals and approved for emergency use in several countries, consisting of two human mAbs obtained by different technologies: REGN10933/casirivimab and REGN10987/imdevimab [8, 40, 59]. Even though mAb REGN10933 is not effective in neutralizing the Beta variant, its efficacy was restored with its use in cocktails [19, 64, 90–92]. The only variant that showed in vitro resistance to this cocktail was Omicron. Due to its resistance and high incidence, the FDA suspended the use of this drug in the USA [8].

Another example of an antibody cocktail approved for emergency use is the one marketed by Eli Lilly. Ly-CoV555/banlanivimab mAb binds to RBD in an up (active) and down (resting) conformation and potentially neutralizes in vitro SARS-CoV-2 [38, 42, 43]. This was the first monoclonal antibody approved for emergency use by the FDA [8]. Subsequently, this antibody was used as a cocktail with CB6/etesevimab, showing better clinical results [43]. However, the antibody combination was not effective against some SARS-CoV-2 VOCs (Table 3), and the FDA suspended its emergency use [6]. Recently, the company Eli Lilly has obtained emergency use approval for a new antibody, called bebtelovimab, which has neutralizing activity against all variants known to date [8, 19, 47, 72].

The AZD7442 cocktail developed by AstraZeneca is composed of two mAbs (AZD 8895 + AZD 1061; tixagevimab + cilgavimab). Both antibodies were obtained from the plasma of convalescent patients and bound to different regions of the S protein in a non-competitive way, thus increasing the chances of virus neutralization. The expectation is to increase the action of the product, lasting from 6 to 12 months after intramuscular administration [25, 62, 63, 90]. This product maintains neutralizing activity against almost all variants evaluated so far, with a reduction against BA.1 sub-variant, that is not the currently predominant variant, maintaining its FDA emergency use license [6].

Other nAbs cocktails have advanced in clinical studies, such as antibodies developed by Brii Bio and TSB Therapeutics, located in China. Both were isolated from convalescent patients with COVID-19. These mAbs were developed to reduce the risk of ADDC and present a prolonged plasma half-life through the YTE mutation. The mAb BRII-196 binds to a highly conserved epitope of the S protein and completely blocks viral entry and neutralizes infection caused by SARS-CoV-2 in cell culture assays. The mAb BRII-198 binds to another epitope of the S protein and presents an additive and synergistic effect when combined with mAb BRII-196 [57, 65, 66].

More recently, a cocktail of potent nAbs developed by Rockefeller University in collaboration with the company Bristol Myers Squibb (C135-LS and C144-LS) from convalescent serum has begun to be evaluated in phase II/III clinical studies. Preclinical studies have demonstrated the effectiveness of the cocktail in inducing high levels of neutralization of SARS-CoV-2, when administered prophylactically and therapeutically, at low doses such as 5.3 mg/kg (mice) and 2 mg/kg (hamster). This cocktail maintains activity against Omicron [69, 70, 90].

The use of monotherapy is not necessarily inferior compared to the use of cocktails, as long as it is based on mAbs with a high resistance barrier and excellent coverage of circulating variants [19, 25]. Due to the existence of several variants of SARS-CoV-2, the development and use of a mAb that can promote the neutralization of all Sarbecoviruses are essential. It may occur by selecting a highly conserved epitope that would be retained functionally, even with the rapid and dynamic evolution of SARS-CoV-2. It has been described that this epitope would be located outside the RBM. A mAb with these characteristics would offer an intrinsically greater barrier to resistance and could be combined with antibodies directed to RBD, which is one of the most mutable and immunogenic regions of the virus but with a high potential for neutralization [53, 55].

Formerly known as VIR-7831, Sotrovimab can be highlighted among the broad-spectrum nAbs, which is an engineered human monoclonal antibody that neutralizes SARS-CoV-2 and several other Sarbecoviruses. This antibody was derived from mAb S309, isolated from a SARS-CoV convalescent in 2003, which binds to the closed and opened states of RBD [78]. In vitro assays showed that the epitope that binds VIR-7831 remains highly conserved among available sequences from circulating viruses with ≥ 99.8% amino acid conservation [35, 44]. Even though it shows activity against the Omicron sub-variants BA.1, BA.4, and BA.5, sotrovimab is no longer authorized to treat COVID-19 in any USA region due to increases in the proportion of Omicron BA.2 sub-variant that is resistant to this antibody [8, 93].

Similarly, a neutralizing antibody known as ADG20 was derived from ADG2 mAb, isolated from memory B cells of a SARS-CoV convalescent patient in 2003. This mAb uses a distinct angle to recognize a highly conserved epitope that overlaps the receptor binding site. It has broad and potent in vitro neutralizing activity against several other class 1 Sarbecoviruses. The prophylactic and therapeutic uses against SARS-CoV-2 were evaluated in animal models, maintaining potent Fc-mediated effector functions, and providing significant protection against SARS-CoV and COVID-19 [51, 53, 55]. The ADG20 mAb, which has a prolonged plasma half-life and presents the potential to provide up to 12 months of protection against COVID-19, is being evaluated in phase II/III, randomized, double-blind, placebo-controlled clinical trials in outpatients with a mild and moderate form of the disease (Tables 2 and 3).

### Resistance of VOCs to nAbs

The literature showed that the Alpha variant is resistant to neutralization by most mAbs targeting the NTD protein S supersite and relatively resistant to some RBD-binding mAbs used for the treatment or prevention of COVID-19. Furthermore, the Beta variant is not only resistant to neutralization by most NTD-binding mAbs but also to the main group of mAbs, more potent and approved for emergency use (Ly-CoV555 alone and in combination with J016 and REGN10933, but not REGN10987), which targets RBM, largely due to the E484K mutation [19, 25, 60, 88].

Some mAbs such as casirivimab, imdevimab, etesevimab, and sotrovimab maintained their ability to neutralize the Delta variant. On the other hand, bamlanivimab does not neutralize this variant [8, 19, 25, 51]. Importantly, the reduction of neutralizing activity in vitro does not always lead to a blockage of therapeutic activity in vivo. For example, Ryu et al*.* demonstrated that the Gamma and Delta variants are resistant to neutralizing activity by CT-P59 in vitro. However, treatment with this antibody in hACE2 transgenic (TG) mice led to improvement of clinical symptoms [67].

Although worrisome, the Delta variant has only 5-point mutations in the S protein, compared to the Omicron variant, which has 32 mutations [94]. A reduction of the activity against the Omicron variant in the cocktails of the companies Regeneron and Eli Lilly was observed through in vitro neutralization assays. The CT-P59 antibody also showed a loss of ability to inhibit the Omicron variant, while the antibodies from the company AstraZeneca showed a small reduction in activity (~12 times) [8, 92, 95].

The genomic surveillance led to the discovery of sublineages of Omicron, including BA.2, which is resistant to most nAbs tested, including sotrovimab, adintrevimab, and amubarvimab/romlusevimab. Most of the nAbs tested failed to neutralize BA.4 and BA.5 sub-variants. However, interestingly, these variants were more sensitive to sotrovimab than BA.2. The sub-variants BA.4 and BA.5 are resistant to most broad nAbs, except for bebtelovimab and cilgavimab. Therefore, in the current scenario of world prevalence of BA.4 and BA.5, bebtelovimab and tixagevimab/cilgavimab are the only effective nAbs [91–93, 95].

The organizations involved in the development and production of the nAbs suspended due to VOCs resistance continue to invest in new nAbs as can be seen in Fig. 5. For instance, Regeneron is investing in 2 new nAbs in clinical trials (REGN 15,160 and REGN 14,256). Celltrion is clinically developing a new antibody, CPT63, alone or in association with CTP59. Shangai Junshi, which is associated with Eli Lilly for the development of etesevimab, now has a new nAb in early clinical development, JS 026.

### Clinical Aspects of Neutralizing Antibodies (nAbs)

The use of nAbs provides a quick standardized number of neutralizing antibodies, capable of generating immediate immunity in a population highly susceptible to severe forms of the disease and who do not respond well to vaccination. Ultimately, their use contributes not only to a reduction in the risk of death but also to a decrease in hospitalization and the burden on the health system [13].

The strategy of using nAbs targeting SARS-CoV-2 was authorized on an emergency basis by the FDA and recommended by the IDSA [13], NIH (National Institute of Health) [14], and WHO [10]. The NIH and the American College of Rheumatology have recommended the use of nAbs for pre-exposure prophylaxis in addition to booster doses of the vaccine against COVID-19 for immunosuppressed patients, as well as for treatment, consolidating this immunotherapy as an important part of the set of measures against COVID-19 in immunosuppressed patients. The recommendation for the use of nAbs in post-exposure prophylaxis is not a consensus among the guidelines due to the different prevalence of VOCs and their sensitivities to nAbs. The use of nAbs has been shown to be an effective and safe strategy for controlling the spread of the virus, reducing clinical symptoms, as well as preventing hospitalizations, and reducing symptom duration in the context of post-exposure prophylaxis.

With the emergence of the Omicron variant and sub-variants and their predominance as the etiology of SARS-CoV-2 infections in the USA, emergency authorizations for casirivimab/imdevimab, bamlanivimab/etesevimab, and sotrovimab were suspended, thus following the last updates on the treatment of mild forms recommended by the NIH [14]. IDSA maintains the recommendation of these three products as a therapeutic option for patients at high risk of progression to severe forms of COVID-19, and WHO recommends just casirivimab/imdevimab for the same clinical scenario, conditioned to the viral sensitivity profile to drugs on an outpatient basis, as soon as the molecular diagnosis is confirmed [10, 13].

The tixagevimab/cilgavimab cocktail (AZD7442), with 300 mg of each nAb for intramuscular use, was recently recommended for pre-exposure prophylaxis in patients with moderate-to-severe immunosuppression. In this case, different from the recommendation for vaccines, it is not necessary to combine the immunosuppressant with the administration of pre-exposure prophylaxis (PrEP). The use of casivirimab/imdevimab was able to prevent symptomatic infection in 81% of the patients and to reduce the evolution to severe forms, hospitalization, and death in 70.4% of cases, and it is still recommended by IDSA for post-exposure prophylaxis in patients unable to produce an induced immune response by vaccination due to immunosuppression, or with contraindication to it, conditioned to the predominant VOCs and their sensitivity profiles [13, 14, 96–98].

Until December 2021, the only therapies recommended in outpatient treatment by the IDSA, WHO, and NIH guidelines were nAbs when the therapeutic set was increased by the antivirals molnupiravir and nirmatrelvir/ritonavir, and by one more nAb, tixagevimab/cilgavimab for pre-exposure prophylaxis, as mentioned above. It is worth mentioning that the nAbs indicated for outpatient treatment should be administered up to the fifth day of symptoms, considering the profile of predominant variants for the appropriate choice of nAb and the high-risk population [10, 13, 14].

On February 11, 2022, the nAb bebtelovimab had its emergency use approved by the FDA, and, now, it is recommended by NIH for persons over 12 years old as a therapeutic alternative to nirmatrelvir/ritonavir and remdesivir only when they are not available or due to clinical concerns, given the potential adverse effects and drug interactions [8, 14, 47, 72].

The capacity of nAbs to provide immediate protection for unvaccinated and vaccine-unresponsive individuals makes this therapeutic option an important strategy to mitigate the new COVID-19 wave’s impact on health systems, while vaccines are under development for VOCs. The fast response to the epidemic would be better if the nAb delivery would not require intravenous infusions. Some approved products use the subcutaneous or intramuscular routes (Table 2), making administration easier, like vaccines, with no need for trained infusion centers. In addition, the rapid transfer of immunity with reduction of viral load that helps to interrupt the chain of viral transmission is clear [13] (Table 2). Indeed, it is expected that intranasal IgG nAbs would block the virus at the nasal cavity and would provide higher nAbs levels in the lung than intravenous infusion. New intranasal formulations of engineered mucosal IgM and IgA nAbs are promising, given their higher respiratory protection than IgG1 against SARS-CoV-2 in mice [86, 99, 100].

There is no formal recommendation by the NIH and IDSA for the use of nAbs in hospitalized patients with severe forms of the disease; however, the WHO has recommended the use of casivirimab/imdevimab in hospitalized patients seronegative for SARS-CoV-2 and where there is a prediction of sensitivity of the virus to the drug based on the phase III Recovery NHS study. This study evaluated 9785 hospitalized patients with COVID-19 and observed that, among seronegative patients for SARS-CoV-2, there was a significant reduction in mortality [10, 14, 97].

## Concluding Remarks and Future Perspectives

The pandemic caused by SARS-CoV-2 resulted in a great negative impact on the world population and health systems. On the other hand, an outstanding effort to accelerate the development of vaccines and new drugs was also observed, highlighting the role of collaboration to minimize risks and uncertainties for all stakeholders, i.e., society, research organizations, manufacturing firms, regulators, and governments. This resulted in the availability of not only vaccines but of nAbs in record time so that different effective prevention and treatment strategies to combat SARS-CoV-2 were made available.

The contribution of nAbs to the fight against the pandemic is clear: its quick availability on the market with the possibility of a rapid response in the containment of possible future new health emergencies until the development and large-scale availability of vaccines; and the wide possibility of indications in different scenarios, such as pre- and post-exposure prophylaxis and outpatient and hospital treatments for individuals most susceptible to severe forms of the disease, such as immunosuppressed patients and those with chronic diseases. The challenge remains to expand access to this effective and safe therapeutic possibility and the rapid clinical availability of new nAbs acting against VOCs.

The synthetic antivirals approved for emergency or compassionate use so far have added therapeutic possibilities for COVID-19, since they offer effectiveness, specificity, and safety that are better established not only by pivotal studies but also with real-world studies involving polymedicated patients, carrying multiple comorbidities, and the elderly. However, to date, none of them has been indicated for prophylaxis, and safety issues are still being evaluated. In comparison, nAbs, which may be combined with other treatments, have a superior safety profile and good results in prevention and treatment, both in clinical trials and real-life studies.

The nAbs scenario remains dynamic, and there are products in various stages of development for prophylactic and therapeutic use, with clinical trials underway, including recent approvals for emergency use. The emergence of new variants threatens the effectiveness of nAbs and other treatments, demanding constant genomic surveillance. From our database, we were able to observe that organizations whose emergency use of their products was prevented due to the low activity against some variants continue to invest in clinical trials of new nAbs to compose their portfolios of products against COVID-19.

Another challenge to overcome regarding this kind of immunotherapy on a large scale is its final cost, especially for low-income countries. Currently, both subcutaneous and intramuscular nAbs are already available. These routes of application make administration easier, like vaccines, and there is no need of trained infusion centers that can bring risks of exposure to the virus. New approaches such as mRNA-encoded antibodies are very promising to increase the scale of nAbs production and reduce costs; however, more studies need to be carried out for safe use.

The current scenario of the pandemic is, unfortunately, not a stable one, raising concerns about the emergence of new variants and the overload of health systems. Thus, there is a constant need to analyze and reanalyze this scenario through technological foresight and literature review, as performed in this study. In this foresight study, the enrichment of data, the quality of the construction of scenarios, and the dissemination of knowledge in different organizational areas were achieved through the multidisciplinary collaborative work of the PG and DG, which has kept the updates of the scenarios, thus following the evolution of these products as an ongoing process. Therefore, although automated algorithms and other technologies do improve and speed up data preparation, analysis and visualization, multidisciplinary collaborative work is fundamental.

The conclusion is that, even with the recent approval of some synthetic drugs and emergence of VOCs, including Omicron, the use of nAbs will continue to be relevant due to its safety profile and the possibility of immediate immunity transfer, especially in polymedicated patients carrying comorbidities as well as immunosuppressed patients (cancer patients, immune-mediated inflammatory diseases, and transplanted patients), as they do not have an adequate immune response to vaccination. Therefore, we suggest that organizations should keep investing in improvements in this technology, specially focusing on broad-spectrum activity against variants and new administration routes. Moreover, the fast and consistent answer for COVID-19 has shown the potential of nAbs for treatment of virus diseases and the knowledge acquired may help the development of new products and contribute to preparedness for emergence of new epidemics.
